# Acute Psychosis Following 1,1-Difluoroethane Inhalation

**DOI:** 10.7759/cureus.5565

**Published:** 2019-09-04

**Authors:** Clara B Novotny, Sarah Irvin, Eduardo D Espiridion

**Affiliations:** 1 Behavioral Medicine and Psychiatry, West Virginia University School of Medicine, Morgantown, USA; 2 Psychiatry, Frederick Memorial Hospital, Frederick, USA

**Keywords:** difluoroethane, inhalant abuse, huffing, acute psychosis

## Abstract

Inhalants are often abused due to their ability to acutely induce feelings of euphoria. Difluoroethane is a toxic lipophilic hydrocarbon that crosses the blood-brain barrier and inhibits the central nervous system. Studies have shown the cardiac, renal, and respiratory effects it has when abused; however, our literature review yielded no previous report of acute psychosis after difluoroethane inhalation. In order to prevent poor outcomes by missed diagnosis, we present a case of difluoroethane-induced acute psychosis.

## Introduction

In the United States, there is a nine percent lifetime prevalence of inhalant abuse [1]. Inhalants are chosen as drugs of abuse as their method of delivery allows avoidance of first pass metabolism to the liver and quick access to the central nervous system. This enhances their capacity to alter mental status. One such inhalant is 1,1-difluoroethane. Commonly used as an aerosol propellant in keyboard cleaners, it is an odorless hydrocarbon that rapidly crosses the blood-brain barrier due to its high lipophilic properties [2-3]. There are several different ways to abuse these drugs including: sniffing, bagging, and huffing [4]. Sniffing involves direct passage from canister to nose. Bagging requires spraying the toxin in a bag and inhaling. Huffing entails soaking a rag with the chemical and inhaling. According to the National Institutes of Health, huffed difluoroethane has caused adverse effects including cardiomyopathy, angioedema, respiratory stridor, and loss of consciousness [3]. While some inhalants have been linked in publication to psychotic symptoms, difluoroethane is not one such inhalant. 

## Case presentation

A 36-year-old female patient was brought to the ED of the local community hospital after she was found unconscious and unresponsive on a grocery store’s bathroom floor. She later admitted to huffing “dusters.” She had consumed an unknown number of computer cleaner canisters to get high. She presented with auditory and visual hallucinations. She reported delusions that she would give birth to Jesus Christ. She was also experiencing visual hallucinations of blurred images. She also reported hearing the voice of “Jesse,” a former boyfriend. She endorsed depressive symptoms with sad mood, anhedonia, fatigue, and feelings of hopelessness. Despite being psychotic and depressed, she had no cognitive impairment, suicidal ideations, or aggressive behavior. Her medical history includes anxiety, hypothyroidism, polycystic ovarian syndrome, endometriosis, and celiac disease. She is prescribed clonazepam 1 mg TID, levothyroxine 50 mcg QD, loratadine 1 tab QD, glucophage 500 mg BID, and lisinopril 5 mg QD by her primary care physician. Urine toxicology screen at presentation was positive for benzodiazepine consistent with home medications and tetrahydrocannabinol consistent with a self-reported recreational use of marijuana. Notably, the patient endorsed a several year history of daily marijuana use of small quantity with no previous psychotic symptoms or adverse psychiatric effects. Due to the new onset of psychosis and melancholic depressive symptoms, the patient was admitted to the inpatient behavioral health unit.

Treatment with olanzapine 20 mg QHS was initiated on the night of her arrival to the behavioral health unit, and on the fifth day she no longer displayed psychotic delusions or hallucinations. Olanzapine was preferred by the physician as the patient displayed insomnia and agitation, and also for its available formulations both PO and IM as needed to manage her psychosis. She tolerated the medication well and noted improvements of both her depressive and psychotic symptoms. She engaged appropriately in individual and group therapy sessions. After a five day hospital stay, the patient was transferred to an inpatient substance abuse program. 

## Discussion

The Diagnostic and Statistical Manual of Mental Disorders, Fifth Edition (DSM-5) identifies inhalant use disorder as “a problematic pattern of use of a hydrocarbon-based inhalant substance leading to clinically significant impairment or distress” with the requirement of meeting two of 12 specific qualifiers relating largely to impairment in daily function [5]. This patient meets these criteria, with both her history of difluoroethane abuse and most recent hospitalization qualifying her. Hydrocarbon inhalants rapidly access the central nervous system because of their lipophilicity. Here, these inhalants stimulate gamma-aminobutyric acid (GABA) receptors, causing inhibition in the central nervous system similar to the effects of ethanol [3]. This can cause euphoria, disorientation, agitation, and impaired judgment. Because euphoria is often experienced, difluoroethane abuse is associated with patients presenting with anhedonia and other depressive symptoms, much like the patient of this case [5]. It provides a rapid high which in turn dissipates within a matter of minutes, making it both highly desirable and highly dangerous for its abusers [6].

Acute adverse effects of difluoroethane inhalation include loss of consciousness, frostbite at mucosal surfaces, rhabdomyolysis, and global myocardial hypokinesis. At the cardiac myocyte, fluorinated hydrocarbons alter potassium and calcium currents, leading to dysrhythmias and prolonged atrial refractory time [4]. These effects cause cardiac injury after acute or chronic use. A case series addressed deaths within a California county which occurred while the decedents were intoxicated with difluoroethane. Cases included loss of consciousness while driving resulting in fatal motor vehicle collisions, death by suicide, and incidental death in otherwise healthy individuals likely involving sudden cardiac compromise [7]. This report demonstrates the immediate danger posed by inhalant abuse.

Inhalant-induced psychotic disorder is defined in the DSM-5 as psychosis with evidence from “history, physical examination, or laboratory findings that the deficits are etiologically related to the effects of inhalant substances” [5]. Nitrous oxide is a substance widely implicated with inhalant-induced psychosis [8-9]. It is known to cause Vitamin B12 deficiency and acute psychosis that resolves with administration of cobalamin [9]. Few studies have been published implicating another inhalant, toluene, to acute inhalant-induced psychosis [10-11]. Options to treat inhalant-induced psychosis include antipsychotics with anticonvulsants, such as haloperidol and carbamazepine respectively, or abstinence and time with resolution of symptoms occurring in two weeks [10]. In one prospective study conducted in Finland, chronic toluene inhalant abuse in adolescence was shown to be independently associated with psychosis later in life [1]. After thorough literature search, we present what is, to the best of our knowledge, the first case report of 1,1-difluoroethane-induced psychosis. Notably, most difluoroethane abusers are adolescents and adult males [4]. This case presents an adult female. Her limited psychotic symptoms resolved with antipsychotic treatment. 

Our patient’s urine toxicology screen tested positive for cannabis, and studies have shown cannabis to induce acute psychosis. Recent literature notes that chronic cannabis use is related to psychosis and schizophrenia later in life [12]. No true confounding variables have been found, but there is an increased risk in individuals with a family history of psychotic disorders and initial use of cannabis at a younger age [13]. Data in another study support the idea that cannabis use does not increase a person’s risk of developing psychosis [14]. It should be noted that our patient’s psychosis acutely improved within days of initiating antipsychotic therapy. This supports the idea that our patient’s acute psychosis was likely not due to cannabis use.

This novel presentation suggests the need for further exploration into diagnosis and treatment of hydrocarbon intoxication. The standard toxicology screen done today does not confirm inhalant abuse; therefore, it is important to consider this as a source when a patient presents with altered mental status and acute psychotic symptoms. Difluoroethane abusing patients are acutely at risk for cardiac failure, and early identification can be life-saving. Thus, clinical suspicion for difluoroethane intoxication should be maintained for patients presenting with unexplained psychosis so as not to miss a crucial and time-sensitive diagnosis.

## Conclusions

This case report highlights the need to consider difluoroethane inhalation for patients presenting to the ED with acute psychosis unexplained by prior diagnosed psychotic disorder and a negative urine toxicology screen. 1,1-Difluoroethane abuse has been associated in publication with mortality by causing impairment leading to trauma, anhedonia resulting in suicide, and cardiac compromise. Appropriate clinical suspicion can allow timely diagnosis which might facilitate proper workup for critical adverse effects including myocardial hypokinesis and dysrhythmias, thus preventing delays in treatment.
